# Effects of a Lower Lipid Diet and Exercise on Cognitive Disturbances with the Induction of Calcineurin 1 and Brain-derived Neurotrophic Factor Expressions in the Hippocampi of Obese Apolipoprotein E Knockout Mice

**DOI:** 10.31662/jmaj.2021-0082

**Published:** 2021-09-21

**Authors:** Shuo Wang, Haicong Li, Mineko Nishijo, Yoshikazu Nishino, Nobuo Kato, Yuji Kasamaki, Tadashi Ueda, Tsugiyasu Kanda

**Affiliations:** 1 Department of Community Medicine, Kanazawa Medical University, Ishikawa, Japan; 2 Department of Geriatrics, China-Japan Friendship Hospital, He Ping Li, Beijing, China; 3 Department of Physiology, Kanazawa Medical University, Ishikawa, Japan; 4 Department of Public Health, Kanazawa Medical University, Ishikawa, Japan

**Keywords:** low-fat diet, exercise, regulator of calcineurin 1, brain-derived neurotrophic factor, ApoE knockout

## Introduction

The regulator of calcineurin 1 (RCAN1) is expressed in neurons and overexpressed in the brains of individuals with both Down syndrome and Alzheimer’s disease (AD) ^(1)^. Its gene expression is enriched in central neurons ^(2)^. RCAN1 can mediate the suppressive effect of energy consumption and regulate obesity control ^(3)^.

RCAN1 might also play an important role in neuronal pathways, and its gene expression is highly correlated with metabolic syndrome ^(3)^. Brain-derived neurotrophic factor (BDNF) is also highly expressed in the brain and plays a crucial role in learning and memory ^(4)^. The reduction of BDNF is associated with decreased cognitive performance in AD.

A high-fat diet (HFD) bidirectionally affects anxiety-related behaviors such that short-term exposure to an HFD reduces anxiety levels, whereas longer exposure to an HFD increases anxiety levels selectively in mice^(5)^. Our previous report showed that the combination of an HFD with exercise maintained over 3 months reduced cognitive dysfunction of obese apolipoprotein E (ApoE)−/−mice ^(6)^.

In this study, we compared the effects of adjusting fat intake and exercise on the cognition and behavioral tasks of ApoE−/− mice. We hypothesized that the induction of RCAN1 and/or BDNF may change cognition and behavior through low-fat intake or exercise. The results of this study may aid people in choosing lifestyle changes regarding fat intake or exercise for protection against neurodegenerative disorders.

## Materials and Methods

### Animals

Forty-eight two-month-old male BALB/c.KOR/Stm Slc-Apoeshl mice (Japan SLC, Inc., Shizuoka, Japan) ^(7)^ were kept in a room at a temperature of 22℃ with a light-dark cycle. The mice were administered either an HFD (45% energy from fat, 24% fat content by weight, 4.73 kcal/g; D12451, Research Diets, supplied by Sankyo Labo Service Corporation, Inc., Japan) or low-fat diet (10% energy from fat, 4.3% fat content by weight, 3.85 kcal/g; D12450H, Research Diets) and drinking water was given *ad libitum*.

All animal experiments followed the Institutional Guidelines of the Kanazawa Medical University (KMU) and the guiding principles of the Physiological Society of Japan. These experiments were approved by the Animal Care Committee of KMU and were performed at the Animal Care Center of KMU.

### Experimental design

Twenty mice were divided into three experimental groups and one control group (*n* = 5 in each group). The mice in the HFD without exercise (HFN) group were fed an HFD (Research Diet, D12451), whereas the changed to low-fat diet without exercise (CFN) group comprised of those fed with a low-fat diet (Research Diet, D12450H). The mice in the third group were fed with the HFD with voluntary exercise (HFE). The fourth group of mice was prepared. The continous low-fat diet without exercise (LFN) group was prepared and analyzed. Mice in the voluntary exercise group ran on SW-15 running wheels (Melquest Ltd., Toyama, Japan). All three groups were given their specific diet and exercise regimen for 3 months, following 3 months of eating the HFD with no exercise. The control group comrised of the mice fed the HFD for 3 months without exercise.

At termination, behavioral tests were performed. All mice were then euthanized using pentobarbital, and cervical dislocation was performed. Body weight was measured and brain tissues were harvested, weighed, and analyzed. Additionally, the hippocampi were immediately harvested for RNA gene expression analysis, as previously performed ^(6)^.

### Laboratory analysis

After the behavioral tests and subsequent euthanization, blood was collected from each heart and centrifuged at 3000 rpm at 4℃ for 10 minutes. Each plasma sample was stored in a refrigerator at 5°C. Total cholesterol (T-CHO), low-density lipoprotein cholesterol (LDL-C), high-density lipoprotein cholesterol (HDL-C), and triglyceride (TG) levels were evaluated at the KMU Hospital Laboratory.

**Behavioral tests**

We performed three behavioral tests with each group. An open field test is an experimental tool for rodents, which evaluates their general locomotor activity, anxiety, and willingness to explore. The novel object recognition test is dependent on the murine spontaneity of exploring objects, specifically novel objects compared with familiar ones. Each mouse was individually habituated to an empty box (40 × 25 × 20 cm) for 3 min and then taken out for a 5-min rest. The Morris water maze test assesses spatial memory in rodents. The details of behavioral tests have been previously reported ^(6)^.

**Statistical analysis**

Statistical analysis was performed using SPSS 22.0, and the results were expressed as the mean ± standard deviation. Comparisons among groups were performed using a one-way analysis of variance followed by Fisher’s least significant difference analysis. The statistical significance level was set at *P* < 0.05.

## Results

### Organ weights

The average body weight of the HFN group was significantly higher than that of the control group, whereas the brain weight of the HFN group was significantly lower than that of the control group (Table 1). The weight of the brain did not significantly differ in the HFE and CFN groups. The brain/body ratio of the HFE group was significantly higher than that of the control and HFN groups. The body weight of the LFN group was significantly lower than the other groups.

**Table 1.** Organ Weights.

Group	Body (g)	Brain (mg)	Brain/Body
Control	32.3±3.5	467.7±16.2	0.0150±0.001
HFN	35.5±2.4*	452.3±27.5*	0.0146±0.0012
HFE	34.1±2.1	462.0±34.2	0.0177±0.0014*†
CFN	33.0±2.3	458.8± 23.0	0.0157±0.0010
LFN	30.0±1.3†	462.4±31.9	0.0154±0.0004

HFE: High-fat diet with exercise; HFN: high-fat diet with no exercise; CFN: changing-fat diet with no exercise. LFN: low-fat diet with no exercise. *P < 0.05 vs. control, †*P* < 0.05 vs. HFN; mean±SD (standard deviation).

### Laboratory analysis

T-CHO and TG levels of the HFN and HFE groups were significantly elevated compared with those of the control group. LDL-C levels in all three groups were significantly decreased compared with the control group. TG and LDL-C levels were significantly lower in the CFN than in the HFN group (Table 2). The TG and LDL-C levels in the LFN group was significantly lower than those in the HFN group.

**Table 2.** Laboratory Data.

Group	T-CHO (mg/dL)	TG (mg/dL)	HDL-C (mg/dL)	LDL-C (mg/dL)
Control	562.6±51.7	87.4±23.1	21.2±4.4	143.6±23.3
HFN	863.5±79.3*	418.5±17.1*	26.0±2.9	55.0±3.4*
HFE	919.2±146.5*	356.2±101.8*†	30.6±9.6	66.8±15.9*
CFN	524.8±104.6	155.0±83.1	21.8±2.2	25.0±4.7*†
LFN	637.0±137.3	98.8±41.0†	18.0±4.1	20.4±3.3*†

T-CHO: Total cholesterol; TG: triglycerides, HDL-C: high-density lipoprotein cholesterol; LDL-C: low-density lipoprotein cholesterol. *P < 0.05 vs. control, †*P* < 0.05 vs. HFN; mean±SD

### mRNA expression

**Figure 1.**
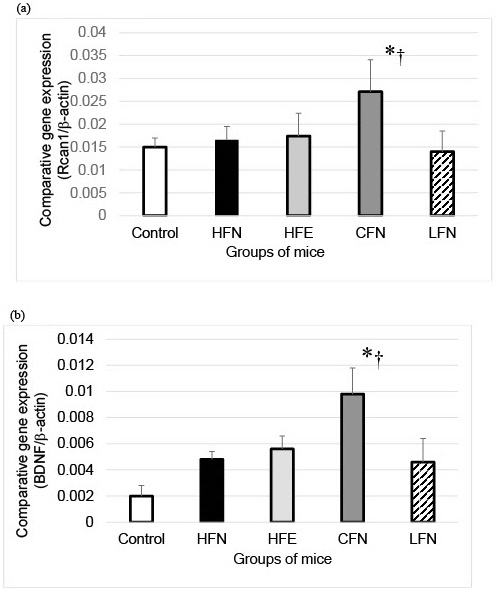
RCAN1 (a) and BDNF (b) expression in hippocampi of each group. Both of RCAN1 and BDNF expressions of CFN were significantly higher than those of the control and HFN. Control: High-fat diet with no exercise for 3 months; HFN: high-fat diet with no exercise for 6 months; HFE: high-fat diet with no exercise for 3 months and then with exercise for 3 months; CFN: high-fat diet with no exercise for 3 months and then low-fat diet with no exercise for 3 months; LFN: low-fat diet with no exercise for 6 months. *P < 0.05 vs. control, †*P* < 0.05 vs. HFN. Error bars represent SD.

RCAN1 mRNA expression in the hippocampi of the CFN group was significantly higher than in those of the other groups (Figure 1a). Additionally, the expression of BDNF mRNA levels in the hippocampi of the CFN group was also significantly higher than in those of the groups (Figure 1b).

### Behavioral tests

**Figure 2.**
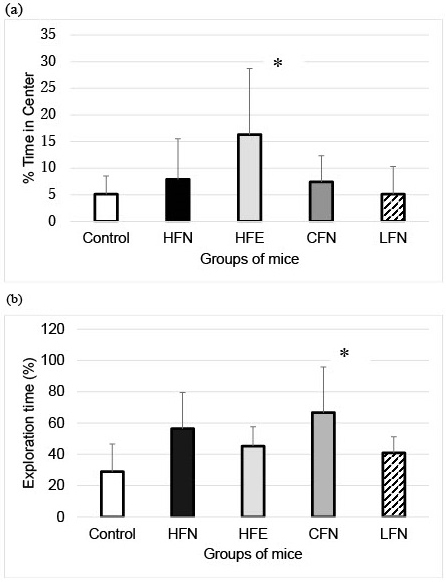
Results of the open field test and the novel object recognition test. The % time of center in open field test as locomotor activity (a) and exploration time of novel object recognition as recognition memory (b) in all four groups. The % time of center of HFE was significantly longer than that of control. The exploration time of CFN was significantly longer than that of control.*P < 0.05 vs. control. Error bars represent SD.

**Figure 3.**
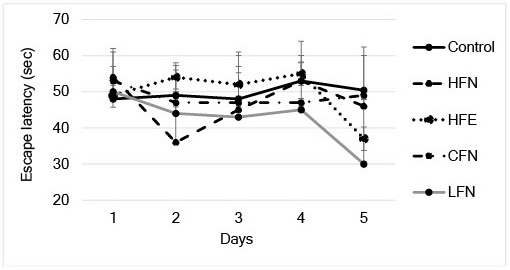
Results of Morris water maze test. There was no difference in time of escape latency in all five groups. Error bars represent SD.

For the open field test, we found that mice in the HFE group spent a significantly longer percent time in the center than those in the control group (Figure 2a). In the novel object recognition test, the exploration time of the CFN was significantly longer than that of the control group (Figure 2b). However, in the Morris water maze test, no significant differences were observed among the groups (Figure 3).

## Discussion

Our findings suggest that changing the diet from high- to low-fat improves recognitive function compared with increased exercise with no change in fat in the diet.

We also identified the induced expression of RCAN1 and BDNF in the hippocampus after the change in lipid intake, even without any change in exercise. Exercise combined with increased lipid intake could play a key role in anxiety function. Additionally, recent studies have shown a link between ApoE and morbid obesity in experimental animals ^(8)^.

Although an HFD can induce functional and structural neuronal plasticity and activate orexin-producing neurons, which prevent depressive behavior, this beneficial function was limited within 4 weeks of initiating the HFD intake ^(9)^. Our results showed that an HFD maintained over 6 months with 3 months of exercise did not improve the cognitive dysfunction of ApoE−/− mice. The effect of short-term exercise may limit the effect of a long term HFD regarding recognitive dysfunction. However, an HFD has been linked to impaired memory ^(10)^. Our results showed that an HFD even with exercise can induce an anxiety state when compared with an HFD without exercise and a low-fat diet without exercise. These results suggest that physiological exercise may not affect neural plasticity in the hippocampus when there are metabolic disturbances induced by a long term HFD. However, a low-fat diet may not have the same effect. Adequate dietary intake, including changes in dietary fat, has important effects on brain dysfunction ^(11)^.

The role of RCAN1 in the brain is controversial. The short-term induction of RCAN1 shows a protective effect in cell survival ^(1)^. In this study, we found induced *RCAN1* mRNA expression in the hippocampus after changing lipid intake even without exercise. The beneficial effect of induction of *RCAN1* by chronic exercise in the hippocampus has not been fully studied yet.

BDNF in the hippocampus plays a key role in the proliferation, differentiation and maintenance of neural integrity. It has been associated with the reduction of cognitive impairment ^(3), (4)^. Our results showed that changing lipid intake induced expression of BDNF in the hippocampus, since higher lipid intake is associated with dementia risks ^(12)^.

A low-fat diet over 3 months improved recognitive function, even without exercise after 3 months of an HFD with no exercise. This was more improvement than was noted with an HFD with 3 months of exercise after 3 months of an HFD with no exercise. An increased amount of fat in the diet can also induce an anxiety state.

## Article Information

### Conflicts of Interest

None

### Sources of Funding

This work was supported by the Grants-in-Aid for Scientific Research of the Ministry of Education, Culture, Sports, Science, and Technology [grant number 17K09330].

### Acknowledgement

We would also like to thank Editage (www.editage.com) for the English language editing.

### Author Contributions

All authors contributed to the concept of this article. WS, TU and TK planned and performed this experiment. HL supervised the experiment. NM, NY and KN supported behavioral tests of mice and conducted this experiment. YS conducted this experiment.
